# Directed Mammalian Gene Regulatory Networks Using Expression and Comparative Genomic Hybridization Microarray Data from Radiation Hybrids

**DOI:** 10.1371/journal.pcbi.1000407

**Published:** 2009-06-12

**Authors:** Sangtae Ahn, Richard T. Wang, Christopher C. Park, Andy Lin, Richard M. Leahy, Kenneth Lange, Desmond J. Smith

**Affiliations:** 1 Signal and Image Processing Institute, University of Southern California, Los Angeles, California, United States of America; 2 Department of Molecular and Medical Pharmacology, David Geffen School of Medicine, University of California Los Angeles, Los Angeles, California, United States of America; 3 Department of Human Genetics, David Geffen School of Medicine, University of California Los Angeles, Los Angeles, California, United States of America; The Hebrew University, Israel

## Abstract

Meiotic mapping of quantitative trait loci regulating expression (eQTLs) has allowed the construction of gene networks. However, the limited mapping resolution of these studies has meant that genotype data are largely ignored, leading to undirected networks that fail to capture regulatory hierarchies. Here we use high resolution mapping of copy number eQTLs (ceQTLs) in a mouse-hamster radiation hybrid (RH) panel to construct directed genetic networks in the mammalian cell. The RH network covering 20,145 mouse genes had significant overlap with, and similar topological structures to, existing biological networks. Upregulated edges in the RH network had significantly more overlap than downregulated. This suggests repressive relationships between genes are missed by existing approaches, perhaps because the corresponding proteins are not present in the cell at the same time and therefore unlikely to interact. Gene essentiality was positively correlated with connectivity and betweenness centrality in the RH network, strengthening the centrality-lethality principle in mammals. Consistent with their regulatory role, transcription factors had significantly more outgoing edges (regulating) than incoming (regulated) in the RH network, a feature hidden by conventional undirected networks. Directed RH genetic networks thus showed concordance with pre-existing networks while also yielding information inaccessible to current undirected approaches.

## Author Summary

An important problem in systems biology is to map gene networks, which help identify gene functions and discover critical disease pathways. Current methods for constructing gene networks have identified a number of biologically significant functional modules. However, these networks do not reveal directionality, that is, which gene regulates which, an important aspect of gene regulation. Radiation hybrid panels are a venerable method for high resolution genetic mapping. Recently we have used radiation hybrids to map loci based on their effects on gene expression. Because these regulatory loci are finely mapped, we can identify which gene turns on another gene, that is, directionality. In this paper, we constructed directed networks from radiation hybrid expression data. We found the radiation hybrid networks concordant with available datasets but also demonstrate that they can reveal information inaccessible to existing approaches. Importantly, directionality can help dissect cause and effect in genetic networks, aiding in understanding and ultimately rational intervention.

## Introduction

Interrogating genome-scale datasets is a necessary step to a systems biology of the mammalian cell [1],[2]. Networks have been constructed using various approaches. In the transcriptome, coexpression networks have been constructed by linking genes whose correlations exceed a selected p-value based on transcript profiling data across different samples [3]. In the proteome, genes can be linked if their corresponding proteins bind each other based on yeast two-hybrid (Y2H) or co-affinity immunoprecipitation assays [4],[5]. Protein-protein interactions can also be ascertained from literature-curated (LC) databases [6],[7]. The Human Protein Reference Database (HPRD) consists of ∼8,800 proteins and ∼25,000 interactions and was constructed using Y2H, co-affinity purification and LC data [6]. Genes can also be linked by virtue of membership of a common pathway [8],[9], an example being the Kyoto Encyclopedia of Genes and Genomes (KEGG) pathway [10]–[12].

Networks constructed using these various approaches are correlated, with some exceptions. While a single dataset often has a large number of false positives and false negatives and reflects only one facet of gene function, accessing multiple independent datasets increases the reliability of gene functional annotation. Integrating diverse gene networks has been shown predictive of loss-of-function phenotypes in yeast [8],[13] and *Caenorhabditis elegans* [9].

Recently transcriptional networks have been constructed using expression data from genetically polymorphic individuals [14]–[16]. This approach allows the identification of quantitative trait loci (QTLs) regulating expression, or eQTLs. Mapping of eQTLs relies on expression perturbations due to naturally occurring polymorphisms. These sequence variants may be lacking in critical pathways because of selective pressure, rendering inaccessible important regions of the genetic network.

A disadvantage of most currently available networks is that it is difficult to infer functional relationships between interacting genes. Consequently, the edges between genes are undirected and have no regulatory hierarchy. This is also true of eQTL networks where, because of limited mapping power, genotype information has been generally ignored and coexpression networks have been constructed instead [17]. Causality between expression and clinical traits has been inferred from eQTL data using conditional correlation measures [18] and structural model analysis [19],[20]. However, this approach has been restricted to a small subset of markers and traits and cannot be easily extended to constructing gene networks.

Radiation hybrid panels have been used to construct high resolution maps of mammalian genomes [21]–[23]. Fragmenting a mammalian genome using radiation yields many more breakpoints than meiotic mapping and hence greatly enhanced resolution. The T31 mouse-hamster hybrid panel was constructed by lethally irradiating mouse cells harboring the thymidine kinase gene (*Tk1^+^*) [22]. These cells were then fused to *Tk1^−^* hamster A23 cells. Selection for the *Tk1^+^* gene using HAT medium resulted in a panel of 100 hybrid cell lines, each of which contained a random sampling of the mouse genome. Mouse autosomal genes retained in a hybrid clone have two hamster copies plus one mouse copy, compared to two copies otherwise.

We recently used the T31 RH panel for high-resolution mapping of QTLs for gene expression [24]. The QTLs regulate expression because of copy number changes and they are therefore called copy number expression QTLs or ceQTLs. We re-genotyped the T31 panel at 232,626 markers using array comparative genomic hybridization (aCGH). The average retention frequency of mouse markers in the panel was 23.9% and the average length of the mouse fragments was 7.17 Mb. We also analyzed the panel using expression microarrays interrogating 20,145 genes.

Using regression, we found 29,769 *trans* ceQTLs regulating 9,538 genes at a false discovery rate (FDR) = 0.4 in the T31 panel. At the same FDR threshold, we also found 18,810 *cis* ceQTLs. Consistent with the average fragment length, a ceQTL was identified as *trans* if >10 Mb from a regulated gene and *cis* otherwise. The 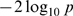 interval for the ceQTLs was <150 kb, thus localizing them to an average of only 2–3 genes.

In this paper we evaluate gene networks constructed from ceQTL mapping. In contrast to undirected networks from meiotically mapped eQTLs and protein binding approaches, the high resolution mapping and dense genotyping of ceQTLs in the RH panel allowed the use of genotype information to construct directed networks. This directionality permits insights that cannot be obtained from undirected networks.

## Results

### A Directed Gene Network from Radiation Hybrids

We previously analyzed a mouse-hamster radiation hybrid panel, T31 [24]. The donor cells were male primary embryonic fibroblasts from the inbred mouse strain 129 and the recipient cells were from the A23 male Chinese hamster lung fibroblast-derived cell line [22]. A total of 99 cell lines from the original panel were available. RH clones with retained autosomal mouse genes in the panel have two hamster copies plus usually one extra mouse copy, compared to two hamster copies otherwise. The variation in gene dosage drives changes in mRNA expression.

Transcript abundance and marker dosage were measured by mouse expression arrays and comparative genomic hybridization arrays (aCGH), respectively. A total of 20,145 transcript levels were assayed by the expression arrays and 232,626 markers by the aCGH. We mapped ceQTLs by regressing the expression array data on the aCGH data. Mouse and hamster genes were detected with comparable efficiency and behaved equivalently in terms of regulation [24].

To construct the RH network, the copy number of each gene was estimated by linear interpolation using the two neighboring aCGH markers. The linear interpolation based estimation is reasonable, considering the high density of aCGH markers.

Measured transcripts were denoted by 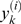, where 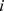 and 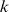 are gene and RH clone index, respectively. The estimated gene copy number was denoted by 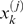 for gene 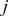 in RH clone 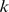. For each ordered pair of genes 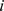 and 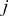, a Pearson correlation coefficient 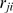 between 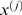 and 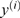 was calculated from the 99 observations. In a linear model 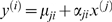, where 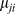 and 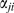 are regression parameters, the correlation coefficient 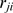 can be viewed as a standardized slope 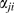 and measures the goodness of fit for the linear model. A significantly large positive 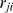 value implies induction and a significantly large negative value implies repression.

Previously, we used an F-statistic, which is monotonic in the absolute value 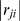 of the correlation coefficient 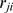, to test for significant association in a context of the linear model [24]. Here we preserved the sign and used the correlation coefficient 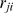 as a test statistic. We found that 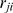 yielded more significant overlaps with other biological datasets than 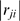 (below). The number of directed edges and number of nodes with ≥1 edge for right-tailed, left-tailed and both-tailed thresholding are shown in Table S1 and Figure S1 (see Methods).

We constructed an adjacency matrix 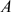 by assigning 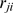 to its 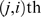 entry, which gives information on whether gene 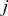 regulates gene 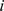, either directly or indirectly. Since 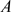 has real number entries and is not symmetric, the network represented by 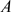 is weighted and directed. We used the correlation coefficients for thresholding and calculated the statistical significance of similarities to existing biological datasets. This is in contrast to transforming the correlation coefficients into FDR (false discovery rate) corrected p-values and then performing statistical thresholding [24]. Our strategy in this study is similar, in spirit, to the integration approach taken in [8],[9] where the reliability of each dataset is measured by comparing with a benchmark dataset.

Since nearly all genes show a copy number increase in a portion of the RH panel, the bulk of genes (94%) also showed a *cis* ceQTL [24]. To remove these *cis* ceQTLs as an artifactual source of edges in the RH network, we omitted all markers within 10 Mb of the gene being considered. Thus, only *trans* ceQTLs were employed in the analysis.

### Overlap with Existing Datasets

We examined the similarity of our network to existing datasets including protein-protein interactions from HPRD (Human Protein Reference Database) [6], the KEGG (Kyoto Encyclopedia of Genes and Genomes) pathway database [10]–[12], Gene Ontology (GO) annotations [25] and a coexpression network obtained from the SymAtlas microarray database of normal mouse tissues [26] (see Methods). We used two different approaches to compare the directed RH and undirected networks. In the first approach, we discarded the edge directions of the RH network and calculated an overlap of undirected edges between the RH and existing networks. It is not uncommon to disregard directions in a network for modeling and analysis purposes [27]–[33] and projecting a directed network onto a space of undirected networks by forgoing information on edge directions seems reasonable. In the second approach, we assumed a hidden directed random network for each undirected existing network and estimated the resulting overlap of directed edges.

#### Undirecting the RH network

To compare the directed RH network and the other undirected networks, we ignored the edge directions in the RH network and calculated the resulting overlap. To test overlap significance, we used a one-sided Fisher's exact test based on a two by two contingency table, replaced with a one-sided chi-square test when the expected values in all table cells exceeded 50 [34] (see Methods). The one-sided Fisher's exact test is equivalent to the hypergeometric test, widely used in Gene Ontology enrichment analysis [35]–[38] and also for evaluating overlap significance between different protein-protein interaction datasets [39]. It is noteworthy that the one-sided chi-square test is closely related to the Bayesian log-likelihood score (LLS) approach to integrating diverse datasets into a single network [8],[9]. That is, the chi-square statistic has a monotonic relationship with the LLS score for evaluating dataset quality (see Text S1).

**Figure 1:**
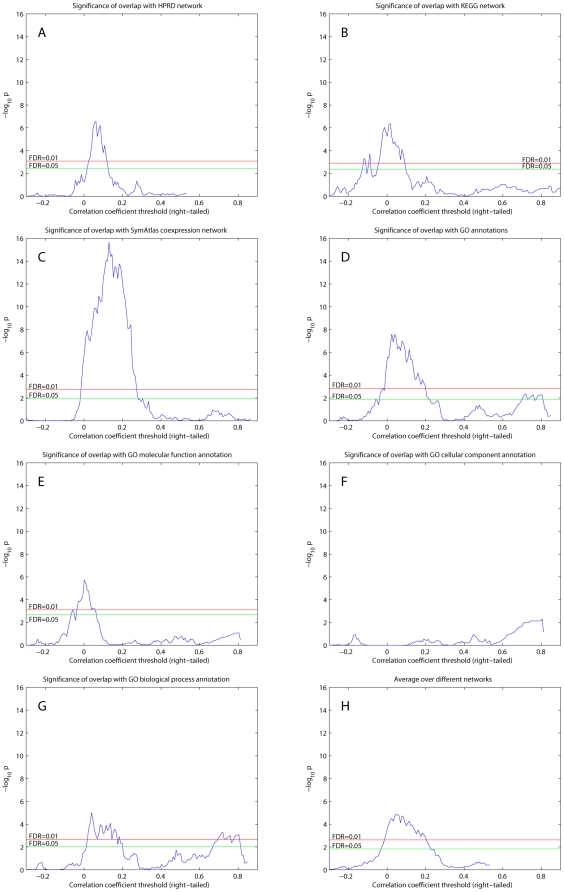
Overlap significance between right-tailed thresholded RH networks and existing datasets. (A) HPRD protein-protein interaction network. (B) KEGG pathway network. (C) SymAtlas coexpression network. (D) GO annotations. (E) GO molecular function annotation. (F) GO cellular component annotation. (G) GO biological annotation. (H) Averaged 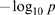 values over results from A to G. One-sided Fisher's exact and chi-square tests used to assess overlap significance.

Figure 1 shows p-values representing overlap significance of the RH network with various datasets for a range of correlation coefficient thresholds (Dataset S1). False discovery rates (FDRs) were calculated following the Benjamini-Hochberg procedure [40]. For correlation coefficient thresholds between about 0 and 0.2, the RH network showed significant overlaps with all datasets (FDR = 0.01) except the GO cellular component annotation network. Although only the biological process annotations from GO were previously used as benchmarks in integrating heterogeneous datasets [8],[9],[13],[41], we also found significant overlap with the GO molecular function annotation.

The existing networks we used for comparison vary in size from 20,957 edges (HPRD network) to 18,754,380 (SymAtlas coexpression network) (see Methods). Nevertheless, the significance of overlaps quantified by p-values was comparable for the different networks (cf. [8],[9]). Figure 1H combines the comparisons of the RH and existing networks by averaging 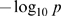 values. The numbers of undirected edges shared with each dataset are shown in Figure S2. The non-monotonic relationships between 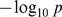 values (Figure 1) and overlap (Figure S2) imply that large 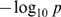 values are likely real and not due to random effects of large numbers of observations. Similarly, the decline in 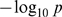 with increasing correlation coefficient thresholds is due to the unavoidable loss of statistical power as edge number decreases. The results suggest that our network possesses biological information relevant to other functional annotations.

**Figure 2:**
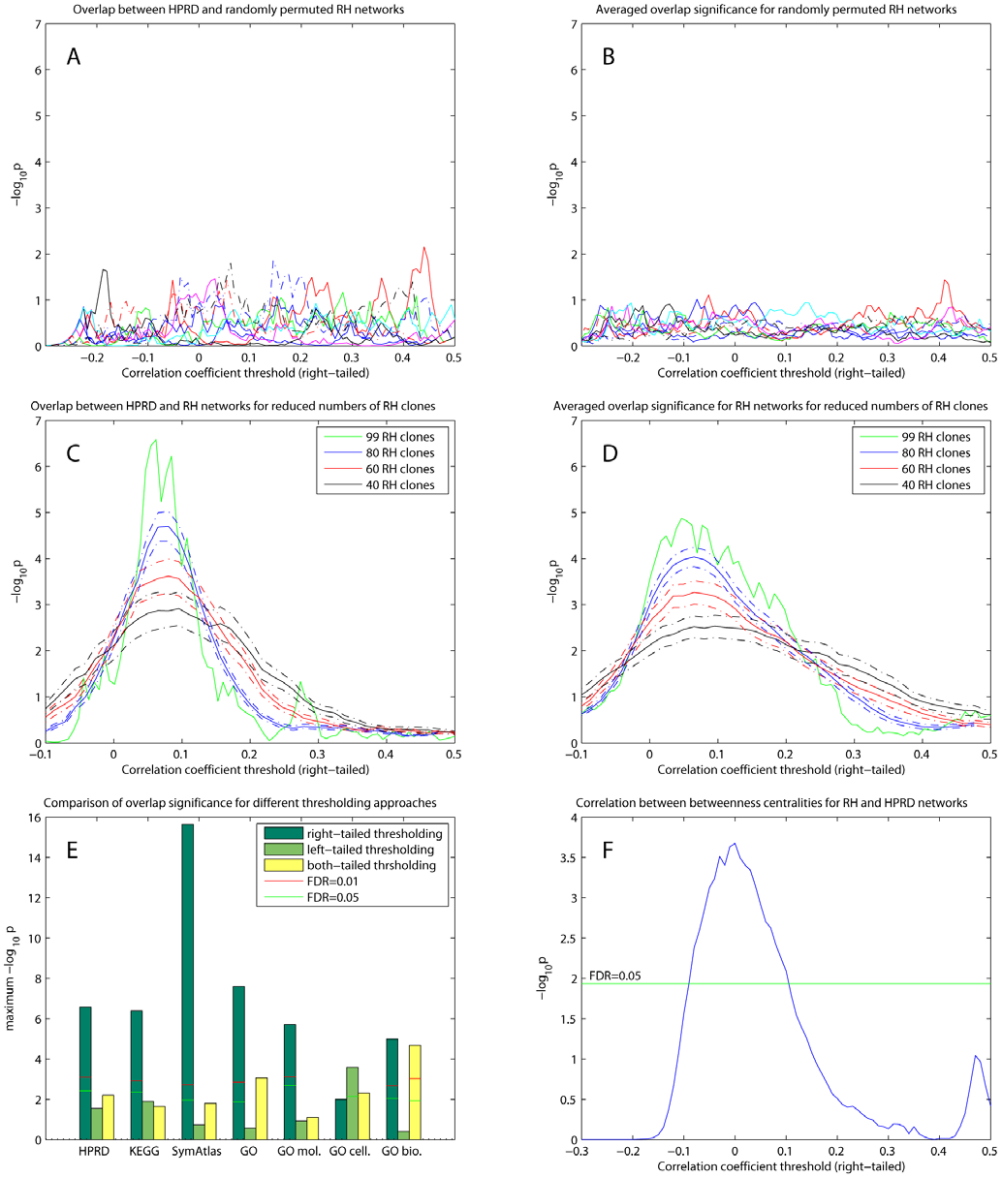
Comparison of RH networks and existing datasets. (A) Overlap between 10 randomly permuted RH networks and HPRD network. The RH networks were constructed from right-tailed thresholding and one-sided Fisher's exact and chi-square tests used to assess significance. (B) Averaged 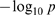 values for overlap between randomly permuted RH networks and different existing datasets (HPRD, KEGG, SymAtlas coexpression, GO, GO-molecular function, GO-cellular component and GO-biological process annotation networks). (C) Overlap between RH networks constructed from a subset of randomly selected RH clones and HPRD network. Mean of overlap significance (solid line) over 50 random subsets shown with standard errors calculated by bootstrapping (dash-dot line). (D) Same as (C) except averaged 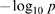 values over different existing datasets. (E) Comparing different thresholding approaches. Maximum 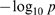 over varying correlation coefficient thresholds shown. (F) Comparing betweenness centralities of RH and HPRD networks. P-values of Spearman correlation coefficients (one-sided, positive direction) between the betweenness centralities of RH and HPRD networks shown.

The maximum overlap significance occurred at low correlation coefficient thresholds between 0 and 0.2 (Figure 1). To test whether this is simply because large thresholds (>0.2) yield too few edges and small thresholds (<0) give too many edges for significant overlap, we randomly permuted the elements of the adjacency matrix for the RH network and repeated the one-sided Fisher's exact and chi-square tests. The permuted network had the same size (number of edges) as the non-permuted RH network. As shown in Figures 2A (overlap with HPRD network) and 2B (overlap significance averaged over existing networks), the permuted networks did not show any significant overlap with the existing datasets (FDR-corrected 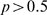). These computational controls imply that the low correlation coefficient thresholds for maximum overlap significance are not simply a statistical artifact.

Next we investigated how the number of RH clones affects the overlap. The sensitivity and resolution of the RH network should improve as the number of RH clones increases. To test this, we randomly selected a subset of the 99 RH clones (40, 60, 80 and 99 clones) and calculated the significance of overlap with the HPRD network using the one-sided Fisher's exact and chi-square tests (Figure 2C). Similarly, Figure 2D shows the 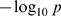 values averaged over the existing datasets. The maximum overlap significance over correlation coefficient thresholds, that is, sensitivity, increased with the number of RH clones (Figures 2C and 2D). However, the correlation coefficient thresholds of maximum overlap significance remained nearly constant between 0 and 0.2 across different numbers of clones (Figures 2C and 2D). This observation implies that the relatively low correlation coefficients of maximum overlap significance may be due to RH network properties orthogonal to existing networks rather than random noise in the array measurements or insufficient RH clones (see Discussion).

#### Hidden directed random network model

We assume that for each undirected network there is a hidden directed random network, modeled as in [42] (see Methods). Since the hidden directed network is not directly observable, we estimated the overlap of directed edges between the directed RH and the unobserved directed networks by a conditional expectation given the undirected existing dataset. P-values representing overlap significance were calculated based on the random network model.

**Figure 3:**
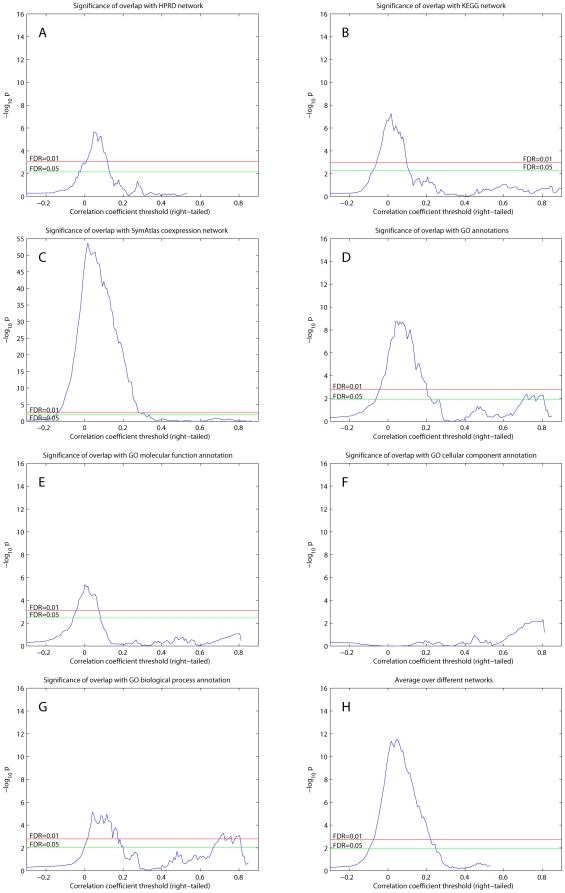
Overlap significance between right-tailed thresholded RH networks and existing datasets, calculated using hidden random directed network models. Same as Figure 1 except that a hidden random directed network was used to model existing undirected networks.

The results of the comparison of the directed RH network and the hidden directed random network are shown in Figure 3. The findings were remarkably similar to those where the directionality of the RH network was discarded (Figures 1) except for scaling factors. The similarity is because the random network model of a hidden directed network, where both directions for an edge are equally probable, does not contain more information than its undirected counterpart. We did not use any topological information on directionality obtained from RH networks since the purpose of the overlap analysis was to explore and validate the RH networks by comparison with independent datasets. In addition, orienting the edges of undirected networks, such as protein-protein interaction networks, is a difficult task since there is no genotype information in these datasets.

### Upregulation Gives More Significant Overlap with Existing Datasets

We examined whether upregulation in the RH data, represented by positive correlation coefficients, 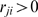, showed a different significance of overlap with existing datasets than downregulation, represented by 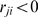. We defined an unweighted adjacency matrix 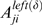 by left-tailed thresholding of the RH data, where 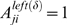 if 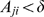 for a given correlation coefficient threshold 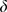, and 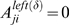 otherwise. This network emphasized downregulation in the RH data. We also defined 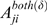 by both-tailed thresholding, where 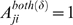 if 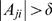, and 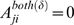 otherwise. This network gave equal weight to up- and downregulation in the RH data and is equivalent to previous datasets produced from F-tests [24]. The unweighted adjacency matrix for right-tailed thresholding is defined as 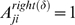 if 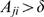, and 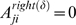, emphasizing upregulation in the RH data.

Unweighted RH networks obtained from left-tailed thresholding, which emphasized downregulation, did not show any significant overlap (FDR-corrected 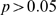) with existing datasets (Figure S3, Dataset S1), except the GO cellular component annotation. Even this significance was modest. Unweighted networks obtained by both-tailed thresholding, which equally weighted up- and downregulation, also did not show any significant overlap (FDR-corrected 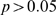) with existing datasets, except the GO biological process annotation (Figure S4, Dataset S1).

Figure 2E compares the maximum significance 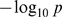 over correlation coefficient thresholds for the different thresholding approaches. Overall, the results suggest upregulation in the RH network yields more significant overlap with existing datasets than downregulation. This may reflect the fact that if a gene represses another gene in *trans* the two protein products are unlikely to co-exist in the cell and hence unlikely to interact. A corollary is that protein binding methods such as yeast two-hybrid and co-affinity immunoprecipitation may miss negative regulatory interactions. Our finding is reminiscent of the observation that interacting protein pairs have significantly higher transcript abundance correlations than chance [43],[44].

### Topological Properties

The overlap analysis based on edge-comparison may fail to capture some indirect interactions or other topologies. We therefore compared the topological properties of the RH and HPRD networks.

The degrees (number of edges for each node, or connectivity) of the weighted (unthresholded) RH and HPRD networks were significantly correlated (Spearman's correlation coefficient = 0.055, 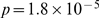). However, the similarity to the HPRD network disappeared when we used absolute values of the correlation coefficients of the RH network in the adjacency matrix, 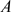 (Spearman's correlation coefficient = −0.0081, 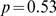). These observations imply that the degree distribution for upregulated but not downregulated edges in the RH network is significantly correlated with the HPRD network. This is consistent with the notion that repressive relationships are not well represented in HPRD.

Next, we compared the betweenness centralities of the RH and HPRD networks. The betweenness centrality measures the total number of nonredundant shortest paths going through each node, representing the severity of bottlenecks in the network [45],[46]. The betweenness centralities of the RH and HPRD networks were significantly correlated (FDR = 0.05) when the right-tailed correlation coefficient thresholds for RH network were between −0.1 and 0.1 (Figure 2F).

We calculated the diameters (average minimum distance between pairs of nodes) of the RH and HPRD networks. The diameter of a giant connected component, consisting of 5,433 nodes with 20,859 undirected edges excepting self-loops, of the HPRD network was 4.13. For the RH network, we considered those 5,433 genes that were in the HPRD network and used a right-tailed threshold of 0.37544, yielding 20,859 undirected edges, to make its size (node and edge numbers) comparable to the HPRD network. The diameter of the RH network was 4.11, close to that (4.13) of the HPRD network.

We also compared the clustering coefficients of the RH and HPRD networks, a measure of local cliqueness [47], but found no significant positive correlation. In summary, the RH network showed similarities with the HPRD network in terms of connectivity, betweenness centrality and diameter, but not cliqueness.

### Essentiality

Previous studies in other networks showed that essentiality is positively correlated with connectivity and betweenness centrality [9], [46], [48]–[56]. However, some authors have questioned the association between essentiality and connectivity, attributing it to dataset bias [6],[57]. We tested whether essentiality is associated with connectivity and betweenness centrality in the RH network.

**Figure 4:**
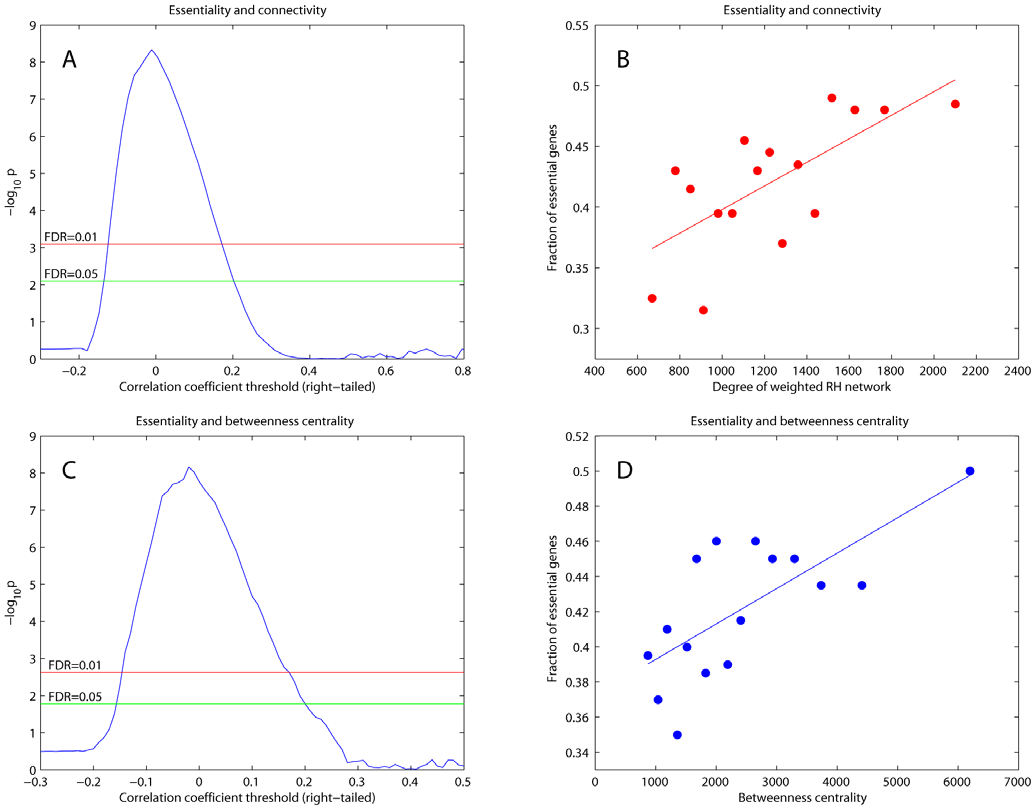
Essentiality, connectivity and centrality in RH networks. (A) P-values for one-sided Wilcoxon rank-sum test assessing whether essential genes have significantly more edges than non-essential. (B) Fraction of essential genes and degree of weighted RH network. (C) P-values for one-sided Wilcoxon rank-sum test assessing whether essential genes have significantly larger betweenness centralities than non-essential. (D) Fraction of essential genes and betweenness centrality of RH network constructed with correlation coefficient threshold of 0.1 by right-tailed thresholding.

Essential genes had significantly more edges than non-essential genes for a range of right-tailed correlation coefficient thresholds from −0.12 to 0.16 (FDR = 0.01) using a one-sided Wilcoxon rank-sum test [34] (Figure 4A). This range is similar to that for significant overlaps with existing datasets. Also, the fraction of essential genes was positively correlated with the degree of the weighted RH network (Pearson's correlation coefficient = 0.70, 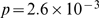) (Figure 4B).

Similarly, essential genes had significantly larger betweenness centralities for a range of right-tailed correlation coefficient thresholds from −0.14 to 0.16 (FDR = 0.01) using a one-sided Wilcoxon rank-sum test (Figure 4C). Figure 4D shows that the fraction of essential genes was positively correlated with betweenness centrality for the RH network constructed from a typically optimal right-tailed correlation coefficient threshold for overlap of 0.1 (Pearson's correlation coefficient = 0.72, 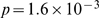).

### Transcription Factors Have More Outgoing Than Incoming Edges

**Figure 5:**
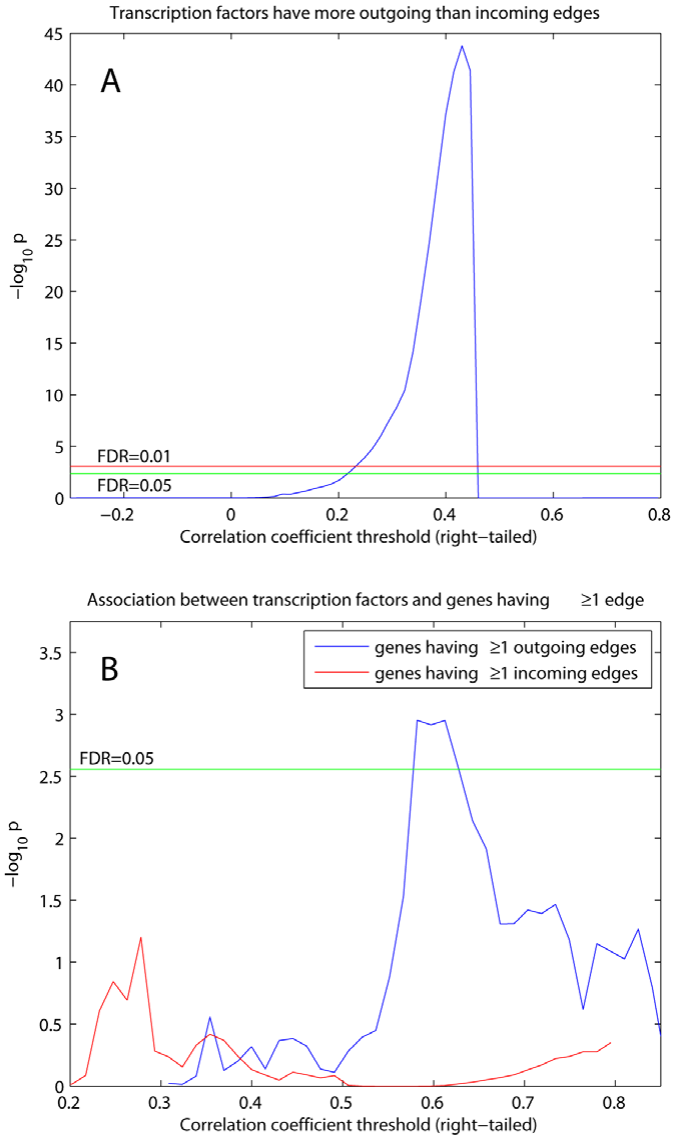
Transcription factors and edge directionality. (A) P-values for one-sided paired signed rank test assessing whether transcription factors have significantly more outgoing than incoming edges. (B) Overlap between transcription factors and genes having ≥1 outgoing or incoming edge. P-values from one-sided Fisher's exact and chi-square tests.

It is natural to suppose that transcription factors would have more outgoing than incoming edges since transcription factors regulate other genes. This proposition cannot be tested in conventional undirected networks, but can be tested in the directed RH network. Using a one-sided paired signed rank test [34] we found that transcription factors had significantly more outgoing edges than by chance (FDR = 0.01) for a range of correlation coefficient thresholds from 0.23 to 0.46 (Figure 5A). We also used a one-sided Fisher's exact and chi-square test to evaluate the association between transcription factors and genes having ≥1 outgoing edge in the RH network. The significance of the association was modest but significant (FDR = 0.05) (Figure 5B). In contrast, the association between transcription factors and genes having ≥1 incoming edge was not significant (FDR = 0.05) (Figure 5B). Together, these results imply that transcription factors are more likely to regulate other genes than be the target of regulation and suggest transcription factors have a privileged role in genetic networks.

## Discussion

We used high resolution mapping of ceQTLs in an RH panel to create a directed genetic network. There was significant overlap with existing networks such as HPRD, KEGG, GO annotation and a SymAtlas coexpression network. The RH network also showed similar topological properties to the HPRD network in connectivity, betweenness centrality and diameter.

The RH network showed maximum significance of overlap with existing networks at relatively low positive correlation coefficient thresholds between 0 and 0.2. The low thresholds were not simply by chance, since randomly permuted RH networks did not show any significant overlap with existing networks. Also, the low values did not seem to be caused by noise in the array measurements or by lack of sufficient numbers of RH clones, since the correlation coefficient thresholds giving maximum overlap significance remained nearly constant for varying clone number, although the sensitivity of overlap increased with the number of clones. This may reflect the orthogonal nature of the RH network compared to existing networks, suggesting the RH approach will yield complementary information on mammalian genetic networks. Novel and replicated edges in the RH network may thus be balanced in the low correlation coefficient threshold range.

The overlap between the RH network and existing interaction networks was greater for edges possessing upregulation than downregulation. This observation may be because the corresponding proteins are unlikely to interact if one gene represses another, since the proteins will not be present in the cell at the same time. It also implies that protein-protein interaction networks may fail to uncover valid edges between genes if they have a repressive relationship.

Previous studies found significant associations of essentiality with connectivity and/or betweenness centrality in protein-protein interaction networks [39], [46], [48]–[52], coexpression networks [53],[56], Bayesian integrated gene networks [9] and transcriptional regulatory networks [46],[50],[54]. Most investigations focused on yeast, worm and fly and there have been only a few studies of mammalian gene networks [6],[9]. Some authors have questioned the association of essentiality and connectivity [6],[57]. Coulomb et al. found that essentiality was poorly related to connectivity when biases in protein interaction databases were taken into account [57]. Yu et al. also found related problems due to bias in a yeast two hybrid dataset [39]. In contrast, the RH network is free of biases that may exist in protein interaction datasets. The significant positive correlation between essentiality, connectivity and betweenness centrality in the RH network adds to the evidence of the centrality-lethality rule in the mammalian setting.

We also showed that transcription factors were likely to have more outgoing rather than incoming edges. While this finding is not unexpected and helps validate the RH network, a recent study using naturally occurring polymorphisms in yeast suggested that transcription factors are no more likely to reside close to eQTLs than chance [58]. The discrepancy between the RH and yeast studies may be because an increase in copy number in the RH cells is a more reliable way to perturb gene networks than naturally occurring alleles. In contrast, polymorphisms may be under selective pressure to minimize disruptions in potentially critical nodes in gene networks, such as transcription factors.

We thresholded the adjacency matrix at different correlation coefficients to compare unweighted RH networks with existing unweighted datasets. However, we chose to leave the RH network weighted rather than finalizing an unweighted form at an optimal threshold. Such an operation is irreversible and would lose information on linkage strength and sign. In other studies, the sensitivity of a coexpression network was limited by thresholding [56] and weighted coexpression networks were more robust than unweighted networks [53]. Indeed, weighted networks are widely used in various applications. In probabilistic integrated gene networks, linkages between genes are represented by weighted sums of log likelihood score (LLS) values [8],[9]. Weighting was also used for a Bayesian gene network [13] and a scientific collaboration network [59]. In addition, weighted coexpression networks have been extensively studied [53],[60] and it is straightforward to incorporate a weighted network into a probabilistic integrated network by a Bayesian LLS approach [8],[9].

We constructed a directed gene network from radiation hybrids and found it concordant with existing networks. We also showed that RH networks have the potential to provide new insights reflecting orthogonal aspects of gene regulation. The RH networks will be refined as more panels, including those available for other species, are analyzed resulting in improved power and sensitivity.

## Methods

### Radiation Hybrid Data

Details on the analysis of the T31 RH panel cells and the preprocessing of aCGH and expression array data can be found in [24]. The microarray and aCGH data have been deposited in NCBI Gene Expression Omnibus (GEO) database under accession number GSE9052.

### Network Construction

The directed RH network was constructed as described in Results. The copy number for each gene was estimated from the aCGH data by linear interpolation as follows. Let 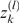 denote the array measurement for aCGH marker 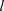 in RH clone 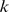. For gene 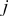, suppose marker 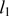 is nearest to the gene from the left on the same chromosome and marker 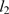 is nearest from the right. The copy number for gene 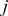 in clone 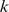 was estimated by 
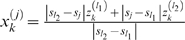
 where 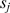, 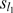 and 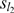 denote the genome coordinates in bp for gene 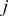 and markers 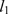 and 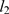, respectively. If gene 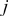 did not have any marker to the left or right on the chromosome, the array measurement for the nearest marker was taken instead.

A protein-protein interaction network was constructed from HPRD (Human Protein Reference Database) [6] by generating an adjacency matrix 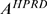, where 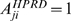 if the proteins corresponding to annotated mouse genes 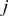 and 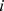 interact with each other and 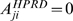 otherwise. Note that 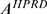 is symmetric and the HPRD network is undirected. The HPRD network had 6,015 nodes and 20,957 undirected edges, excepting self-loops.

A network was constructed from the KEGG (Kyoto Encyclopedia of Genes and Genomes) pathway database [10]–[12] by generating an adjacency matrix 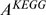 such that 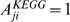 if genes 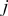 and 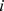 participated in the same pathway and 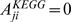 otherwise. The KEGG pathway network had 1,629 nodes and 139,664 undirected edges except self-loops.

A network was constructed from the GO (Gene Ontology) database [25] by generating an adjacency matrix 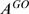 where 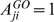 if genes 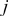 and 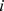 belong to a common GO term and 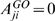 otherwise. Only GO terms with ≤200 genes were considered. Similarly, 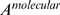, 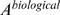 and 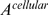 were constructed considering only the GO molecular function terms, GO biological process terms and GO cellular component terms, respectively. The undirected GO, GO-molecular function, GO-biological process and GO-cellular component networks had 10,442 nodes with 786,928 edges, 7,745 nodes with 359,006 edges, 7,653 nodes with 404,641 edges and 3,509 nodes with 140,904 edges, respectively, excepting self-loops. All edges were undirected.

We constructed an mRNA coexpression network from the publicly available SymAtlas microarray database [26]. This database contains transcript profiling data from 61 normal mouse tissues. The Pearson's correlation coefficients of mRNA expression across the mouse tissues were calculated and an adjacency matrix 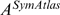 was generated by right-tailed thresholding the correlation coefficients with 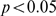. The SymAtlas coexpression network had 15,190 nodes and 18,754,380 undirected edges.

### Overlap Significance Using Undirected RH Network

The significance of overlap between the RH network obtained from thresholding and, for example, the HPRD network was tested as follows.

First, for a given threshold 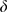, the adjacency matrix 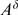 of an unweighted RH network was constructed where 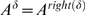 for right-tailed thresholding, 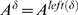 for left-tailed thresholding and 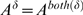 for both-tailed thresholding (see Results). Second, for a comparison with the unweighted HPRD network, the adjacency matrix 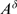 was forced to be symmetric by constructing a symmetric matrix 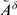 for an undirected RH network such that 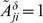 if 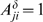 or 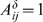, and 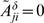 otherwise. Third, a two by two contingency table was built showing the relationship between 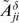 (1 or 0) and 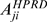 (1 or 0), where only pairs of genes in common to both networks are taken. In addition, for all networks, only gene pairs separated by at least 10 Mb on a chromosome or on different chromosomes were selected. This requirement was imposed to remove possible biases due to copy number effects of a gene's own dosage in the RH network and to ensure gene pairs were in *trans*. Fourth, an overlap was defined as the number of gene pairs such that both 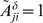 and 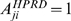. Then a one-sided Fisher's exact test was performed to evaluate whether the overlap was significant and calculate a p-value. If the expected values in all table cells exceeded 50, a one-sided chi-square test was used to reduce computational cost.

We similarly calculated the significance of overlaps with the KEGG pathway network, the SymAtlas coexpression network and the GO annotations.

#### Randomized RH network

We randomly permuted the elements of the weighted and directed adjacency matrix 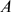 that correspond to gene pairs in *trans* and performed the overlap significance test (above).

#### RH network from a subset of clones

We randomly selected 40, 60 or 80 RH clones out of 99 and constructed an adjacency matrix (see Results) using measured transcripts and copy numbers for the selected clones. Then we calculated the significance of overlap with existing databases (above). We repeated this 50 times for a fixed number of clones.

### Overlap Significance Using Hidden Directed Random Network Model

For each existing undirected dataset, for example, the HPRD network, we assume there is a hidden directed random network with adjacency matrix 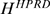, whose elements 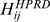 are independent Bernoulli random variables with success probability 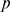. We suppose only the undirected version 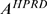 is observed, where 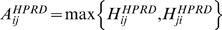 (recall only off-diagonal elements are considered, that is, 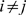). Then 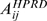 for 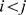 are independent Bernoulli random variables with success probability 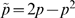. Therefore, using an empirical success probability 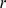, the ratio of 1's to the total in 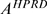, the success probability of the hidden directed random network can be estimated as 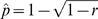.

The overlap between the unweighted (thresholded) directed RH network, represented by 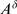, and the hidden directed HPRD network is given by 
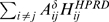
. However, the overlap is not directly observable and instead we calculate the conditional expectation given 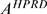. Since 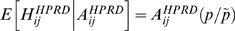, it can be seen that
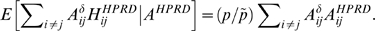



Ignoring the constant scaling factor without loss of generality, we define an overlap as 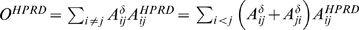 (recall that 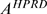 is symmetric whereas 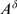 is not). To test whether an observed overlap 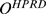 is greater than chance, we calculate a p-value as the probability of the overlap being greater than or equal to the observed value assuming the HPRD network is a random network as described above,
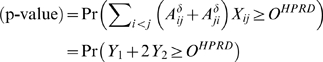
where 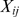 are independent Bernoulli random variables with success probability 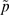 and 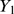 and 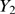 are independent binomial random variables, 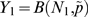 and 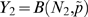, with 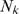 being the number of unordered pairs 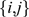 such that 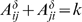 for 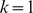 or 2. To reduce the computation cost, 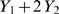 is approximated using the normal distribution when 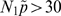, 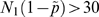, 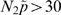 and 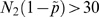.

### Topological Measures

The node degree of the undirected, weighted adjacency matrix 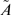 where 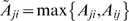 was calculated by 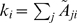. Similarly, the degree of the HPRD network was calculated by 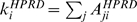. Then we calculated the Spearman's correlation coefficients between 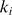 and 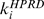.

The betweenness centralities and clustering coefficients of the RH adjacency matrix 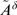 and the HPRD adjacency matrix 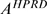 were calculated using MatlabBGL (http://www.stanford.edu/~dgleich). When we calculated the betweenness centrality of the RH network, we used a subgraph by taking nodes that were in the HPRD network to reduce computational cost. Then the Spearman's correlation coefficients between the betweenness centralities and also between clustering coefficients for RH and HPRD were calculated.

### Essentiality and Connectivity and Betweenness Centrality

We obtained a list of 1,409 essential genes and 1,979 nonessential genes from the Mouse Genome Database [6],[61]. Those 3,388 genes were sorted by degree and binned into successive bins of 200 genes and the correlation between mean degree and fraction of essential genes calculated [9]. The betweenness centrality for the RH network was calculated from 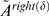, taking a subgraph consisting of a total of 3,388 genes of interest to reduce computational cost and 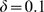. Similarly, the 3,388 genes were sorted by betweenness centrality and the significance of correlation between the mean betweenness centrality and the fraction of essential genes tested.

### Transcription Factors and Edge directionality

We obtained a list of 1,053 transcription factors by finding genes whose GO description includes a word “transcription.” The number of outgoing edges was calculated by 
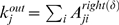
 for gene 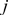 and the number of incoming edges by 
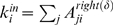
 for gene 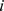. We used a one-sided paired signed rank test [34] to assess whether transcription factors have larger 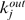 than 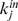.

### URL

The network data are available at http://labs.pharmacology.ucla.edu/smithlab/RHnetwork.html

## Supporting Information

Text S1 Relationship between one-sided chi-square test and Bayesian log-likelihood score (LLS) method
(0.08 MB PDF)

Table S1 Size of RH network constructed from right-tailed, left-tailed and both-tailed thresholding approaches.
(0.06 MB PDF)

Figure S1 Size of RH network. (A) Number of nodes with nonzero degree for RH network constructed from right-tailed thresholding. (B) Number of directed edges for RH network constructed from right-tailed thresholding. (C) Number of nodes with nonzero degree for RH network constructed from left-tailed thresholding. (D) Number of directed edges for RH network constructed from left-tailed thresholding. (E) Number of nodes with nonzero degree for RH network constructed from both-tailed thresholding. (F) Number of directed edges for RH network constructed from both-tailed thresholding.
(0.21 MB TIF)

Figure S2Overlap between RH network constructed from right-tailed thresholding and existing datasets. Same as Figure 1, except number of overlapping undirected edges shown instead of 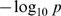.(0.26 MB TIF)

Figure S3 Significance of overlap between RH network constructed from left-tailed thresholding and existing datasets. Same as Figure 1 except left-tailed thresholding.
(0.25 MB TIF)

Figure S4 Significance of overlap between RH network constructed from both-tailed thresholding and existing datasets. Same as Figure 1 except both-tailed thresh-olding.
(0.28 MB TIF)

Dataset S1 Significance of overlap between RH network and existing datasets. Figures 1, S3 and S4 based on this dataset using one-sided Fisher's exact and chi-square tests. Expected and observed overlap and corresponding p-values shown.
(0.78 MB XLS)
