# Supplementary material for: Directed Mammalian Gene Regulatory Networks Using Expression and Comparative Genomic Hybridization Microarray Data from Radiation Hybrids
Source: PLoS Comput Biol. 2009 Jun 12;5(6):e1000407. doi: 10.1371/journal.pcbi.1000407 (PMC2690838; doi:10.1371/journal.pcbi.1000407)
Supplement: Text S1 — Relationship between one-sided chi-square test and Bayesian log-likelihood score (LLS) method (0.08 MB PDF) [file pcbi.1000407.s001.pdf]

Text S1. Relationship between one-sided chi-square test and Bayesian log-likelihood score (LLS) method

Here we show that the one-sided chi-square test used for evaluating the significance of the overlap between the RH network and other existing datasets and the Bayesian log-likelihood score (LLS) approach used for integrating diverse datasets [1,2] are closely related. The Fisher's exact test was used instead of the chi-square test when the expected value in a cell of the contingency table was ≤ 50 (see Methods). However, the chi-square test approximates the Fisher's exact test and useful insights into the overlap analysis are obtained through examining the chi-square test.

Suppose a reference and a test network, both of which are unweighted, and the following contingency table are given.

	Linked edges in test network	Non-linked edges in test network	Total
Linked edges in reference network	a	b	a+b
Non-linked edges in reference network	c	d	c+d
Total	a+c	b+d	a+b+c+d

A chi-square statistic without any correction is given by

$$\chi^2 = \frac{(ad-bc)^2(a+b+c+d)}{(a+b)(c+d)(b+d)(a+c)}. \qquad (1)$$

We used this statistic for evaluating the significance of the overlap between the RH and other existing networks. A log-likelihood score (LLS) is defined as

$$LLS = \log \frac{\dfrac{P(\text{linkage in reference network} \mid \text{linkage in test network})}{P(\text{no linkage in reference network} \mid \text{linkage in test network})}}{\dfrac{P(\text{linkage in reference network})}{P(\text{no linkage in reference network})}} = \log \frac{a(c+d)}{c(a+b)} \qquad (2)$$

and was used for measuring the data quality of the test network compared to the gold standard reference network in a modified Bayesian framework [1,2]. We impose a constraint that $LLS \geq 0$. Indeed, only the datasets with $LLS \geq \log 1.5$ were used in [1,2]. By combining (1) and (2), one can rewrite χ^2 using LLS :

$$\chi^2 = (a+b+c+d)\frac{(a+c)(c+d)}{(a+b)(b+d)}\left(1 - \frac{a+b+c+d}{(a+b)\exp(LLS)+(c+d)}\right)^2 .$$

The chi-square statistic χ^2 is a bijective function of LLS for $LLS \geq 0$. Therefore, the chi-square statistic has a monotonic relationship with the log-likelihood score (LLS).

[1] Lee I, Date SV, Adai AT, Marcotte EM (2004) A probabilistic functional network of yeast genes. Science 306: 1555-1558.

[2] Lee I, Lehner B, Crombie C, Wong W, Fraser AG, Marcotte EM (2008) A single gene network accurately predicts phenotypic effects of gene perturbation in *Caenorhabditis elegans*. Nat Genet 40: 181-188.
