# Supplementary material for: Directed Mammalian Gene Regulatory Networks Using Expression and Comparative Genomic Hybridization Microarray Data from Radiation Hybrids
Source: PLoS Comput Biol. 2009 Jun 12;5(6):e1000407. doi: 10.1371/journal.pcbi.1000407 (PMC2690838; doi:10.1371/journal.pcbi.1000407)
Supplement: Table S1 — Size of RH network constructed from right-tailed, left-tailed and both-tailed thresholding approaches. (0.06 MB PDF) [file pcbi.1000407.s002.pdf]

**Table S1. Size of RH network constructed from right-tailed, left-tailed and both-tailed thresholding approaches.**

| Correlation coefficient threshold | Right-tailed thresholding | | Left-tailed thresholding | | Both-tailed thresholding | |
|---|---|---|---|---|---|---|
| | Num. of nodes with ≥1 edge | Num. of directed edges | Num. of nodes with ≥1 edge | Num. of directed edges | Num. of nodes with ≥1 edge | Num. of directed edges |
| 0.9 | 10 | 9 | 20,145 | 401,775,989 | 10 | 9 |
| 0.8 | 133 | 132 | 20,145 | 401,775,866 | 133 | 132 |
| 0.7 | 520 | 508 | 20,145 | 401,775,490 | 520 | 508 |
| 0.6 | 1,848 | 1,992 | 20,145 | 401,774,006 | 1,848 | 1,992 |
| 0.5 | 6,605 | 10,199 | 20,145 | 401,765,799 | 6,616 | 1,0263 |
| 0.4 | 18,463 | 128,551 | 20,145 | 401,647,447 | 18,733 | 135,237 |
| 0.3 | 20,145 | 1,622,382 | 20,145 | 400,153,616 | 20,145 | 1,994,113 |
| 0.2 | 20,145 | 13,865,830 | 20,145 | 387,910,168 | 20,145 | 2,1761,977 |
| 0.1 | 20,145 | 69,376,471 | 20,145 | 332,399,527 | 20,145 | 132,863,571 |
| 0 | 20,145 | 198,713,118 | 20,145 | 202,526,457 | 20,145 | 401,239,575 |
| -0.1 | 20,145 | 338,288,898 | 20,145 | 63,487,100 | - | - |
| -0.2 | 20,145 | 393,879,851 | 20,145 | 7,896,147 | - | - |
| -0.3 | 20,145 | 401,404,267 | 20,036 | 371,731 | - | - |
| -0.4 | 20,145 | 401,769,312 | 4232 | 6,686 | - | - |
| -0.5 | 20,145 | 401,775,934 | 71 | 64 | - | - |
