# Supplementary figures and images for: Directed Mammalian Gene Regulatory Networks Using Expression and Comparative Genomic Hybridization Microarray Data from Radiation Hybrids
Source: PLoS Comput Biol. 2009 Jun 12;5(6):e1000407. doi: 10.1371/journal.pcbi.1000407 (PMC2690838; doi:10.1371/journal.pcbi.1000407)

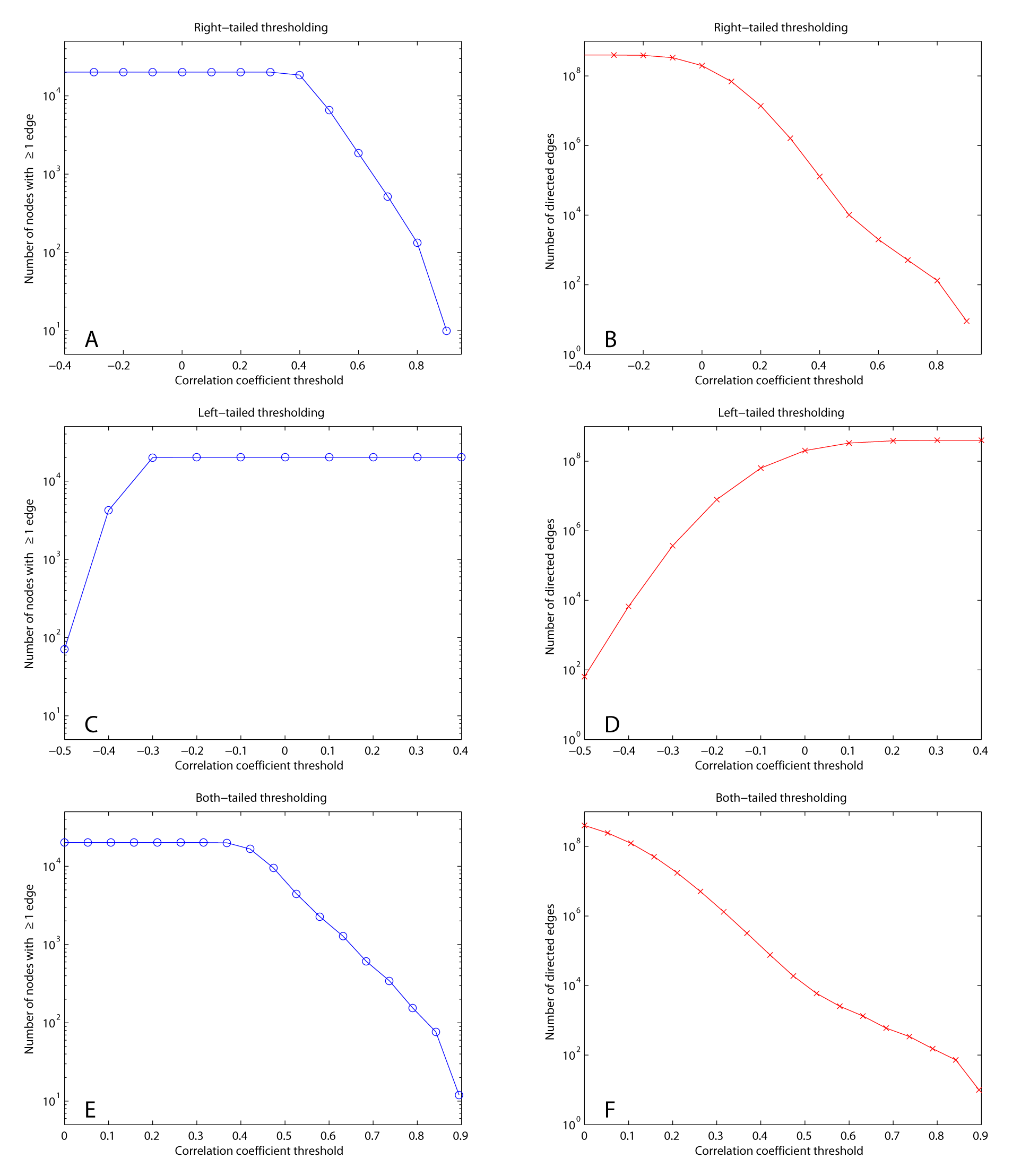

Supplement: Figure S1 — Size of RH network. (A) Number of nodes with nonzero degree for RH network constructed from right-tailed thresholding. (B) Number of directed edges for RH network constructed from right-tailed thresholding. (C) Number of nodes with nonzero degree for RH network constructed from left-tailed thresholding. (D) Number of directed edges for RH network constructed from left-tailed thresholding. (E) Number of nodes with nonzero degree for RH network constructed from both-tailed thresholding. (F) Number of directed edges for RH network constructed from both-tailed thresholding. (0.21 MB TIF) [file pcbi.1000407.s003.tif]

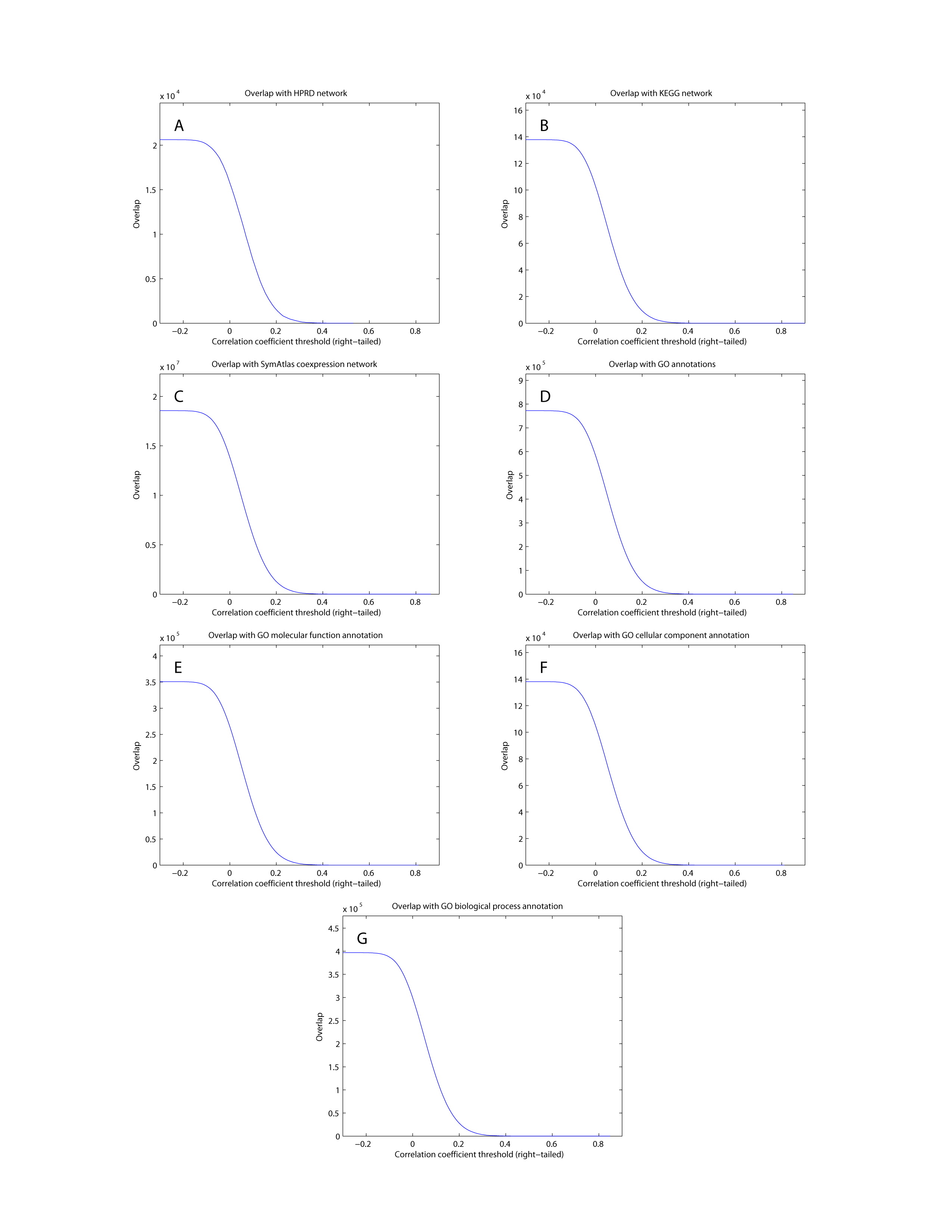

Supplement: Figure S2 — Overlap between RH network constructed from right-tailed thresholding and existing datasets. Same as Figure 1, except number of overlapping undirected edges shown instead of . (0.26 MB TIF) [file pcbi.1000407.s004.tif]

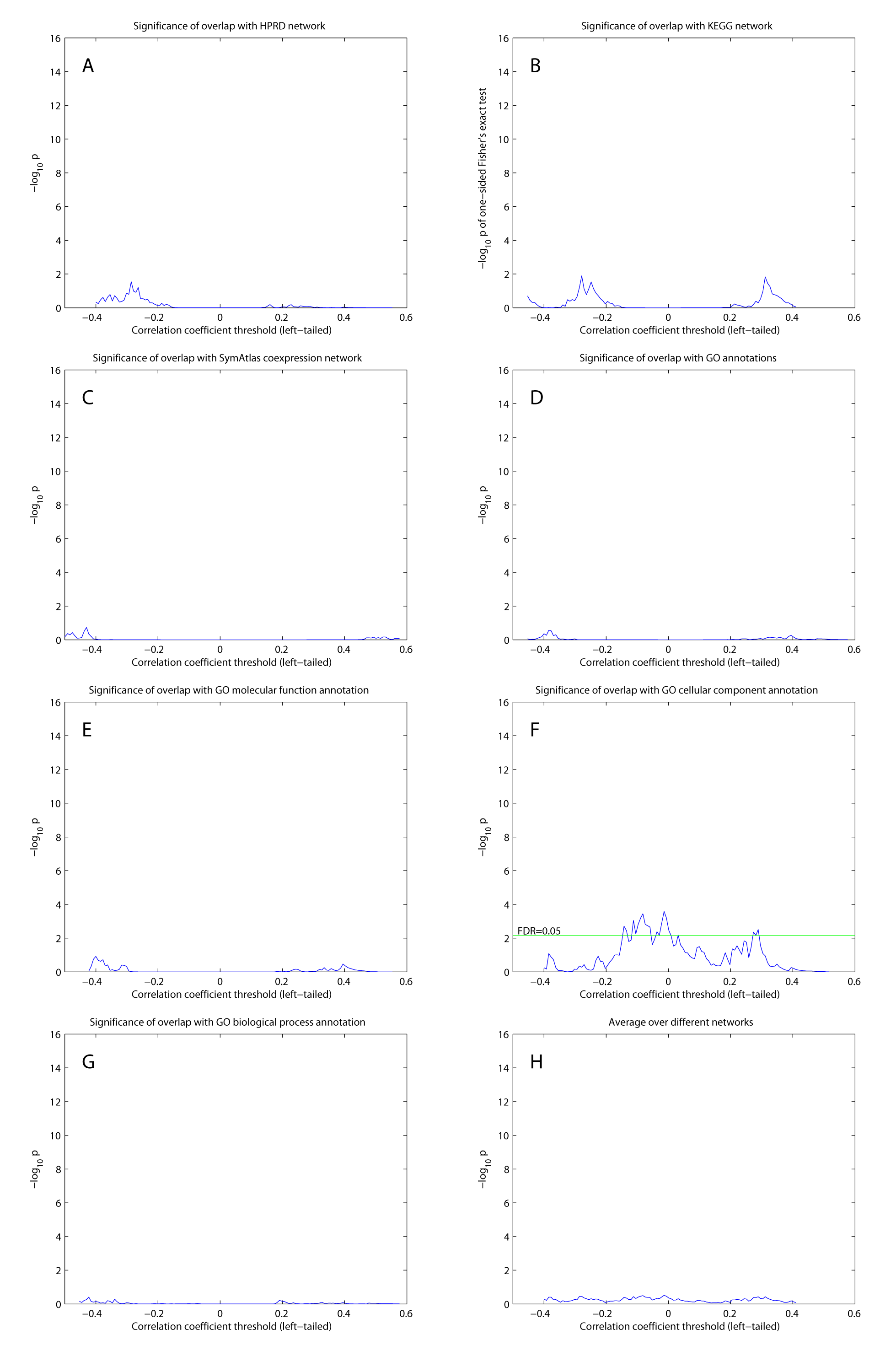

Supplement: Figure S3 — Significance of overlap between RH network constructed from left-tailed thresholding and existing datasets. Same as Figure 1 except left-tailed thresholding. (0.25 MB TIF) [file pcbi.1000407.s005.tif]

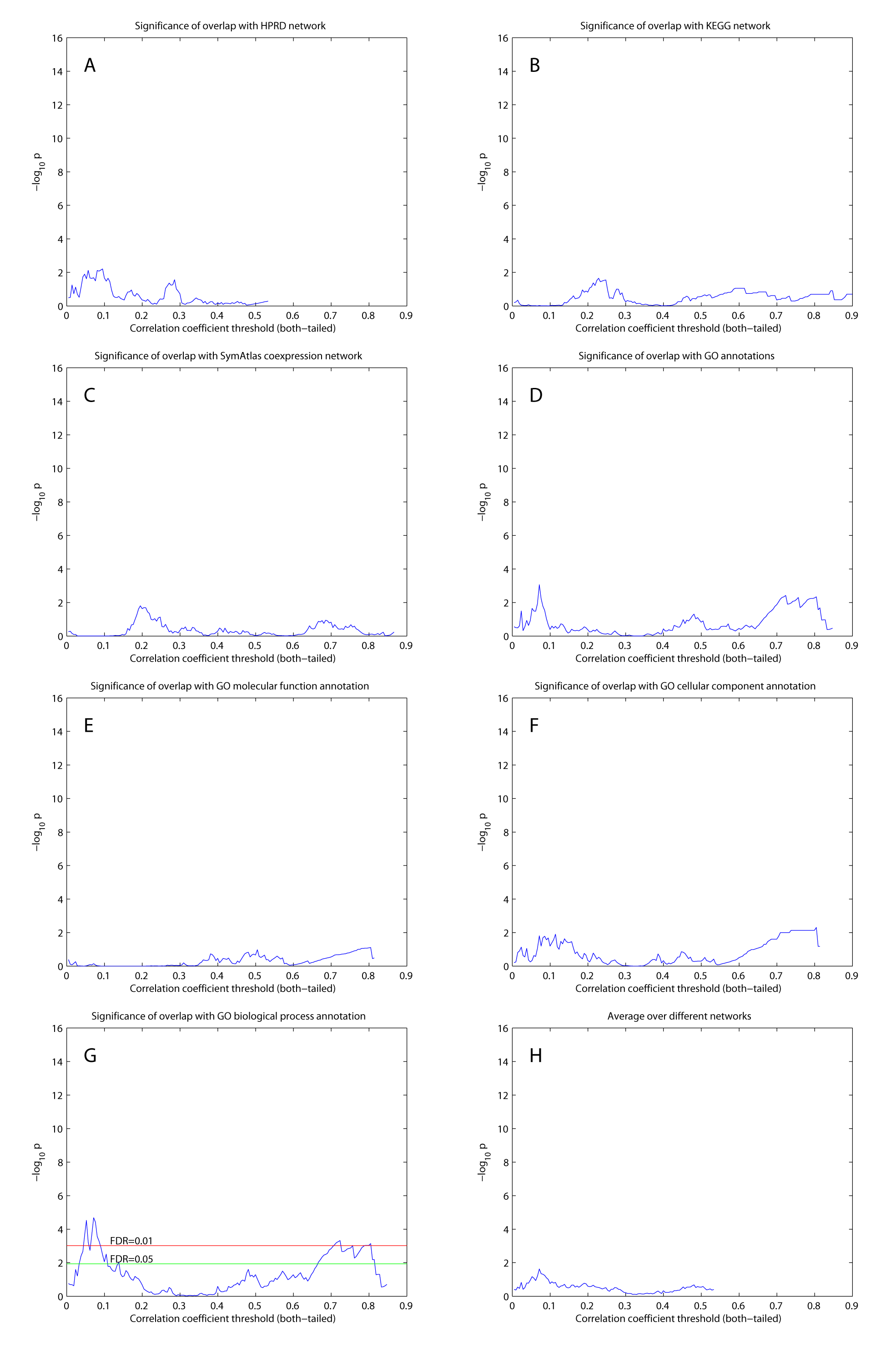

Supplement: Figure S4 — Significance of overlap between RH network constructed from both-tailed thresholding and existing datasets. Same as Figure 1 except both-tailed thresh-olding. (0.28 MB TIF) [file pcbi.1000407.s006.tif]
